# Characteristics, outcome and treatments with cranial pachymeningitis: A multicenter French retrospective study of 60 patients

**DOI:** 10.1097/MD.0000000000011413

**Published:** 2018-07-27

**Authors:** Arsene Mekinian, Lucas Maisonobe, Latifatou Boukari, Cléa Melenotte, Benjamin Terrier, Xavier Ayrignac, Nicolas Scheinlitz, Damien Sène, Mohamed Hamidou, Amadou Konaté, Philippe Guilpain, Noémie Abisror, Etienne Ghrenassia, Florence Lachenal, Ramiro Cevallos, Richard Roos-Weil, Le Thi Huong Du, Francois Lhote, Claire Larroche, Jean-Francois Bergmann, Sébastien Humbert, Jean Baptiste Fraison, Jean Charles Piette, Loïc Guillevin, Robin Dhote, Zahir Amoura, Julien Haroche, Olivier Fain

**Affiliations:** a AP-HP, Hôpital Saint Antoine, Service de Médecine Interne et Inflammation-Immunopathology-Biotherapy Department (DHU i2B), Paris; b AP-HP, Hôpital Jean Verdier, Service de Médecine Interne, Bondy; c Département de Médecine Interne, CHU de la Timone, Aix-Marseille Université, AP-HM, Marseille; d Université Paris Descartes, Paris; e AP-HP, Hôpital Cochin, Centre de Référence des Maladies Auto-immunes et Systémiques Rares, Service de Médecine Interne, Paris; f Département de Neurologie, Hôpital Gui de Chauliac, CHU de Montpellier; g Département de Médecine Interne, GH Saint-Louis Lariboisière Fernand Widal; h Université Paris Diderot, Paris; i Service de Médecine Interne, CHU Hôtel-Dieu, Nantes; j Service de Médecine Interne et Vasculaire, CHU Montpellier, Montpellier; k Service de Médecine Interne, Hôpital Pierre Oudot, Bourgoin Jailleu; l Service de Médecine Interne, Clinique Sainte Anne, rue Philippe Thyss, Strasbourg; m Service de Neurologie, Centre Hospitalier Compiègne, Compiègne; n Service de Médecine Interne, Hôpital Pitié Salpetrière, Université Paris, APHP, Paris; o Université Pierre et Marie Curie, Paris, UPMC; p Centre National de Référence des Maladies Auto-immunes et Systémiques Rares; q Service de Médecine Interne, Hôpital Delafontaine, Saint Denis; r Service de Médecine Interne, Université Paris, AP-HP, Avicenne, Bobigny; s Service de Médecine Interne, CHU Jean Minjoz, Besançon; t Service de Médecine Interne, Hôpital Pitié Salpetrière, Université Paris, APHP, Paris, France.

**Keywords:** Erdheim-Chester disease, granulomatosis with polyangiitis, idiopathic pachymeningitis, IgG4-related disease, pachymeningitis, sarcoidosis

## Abstract

The aim of this study was to determine the characteristics, treatment, and outcome according to each etiology of pachymeningitis.

We conducted a retrospective multicenter French nationwide study between 2000 and 2016 to describe the characteristics, outcome, and treatment of pachymeningitis.

We included 60 patients (median age 55.5 years; interquartile range [IQR] 30–80, female/male ratio 0.43). Neurologic signs were present in 59 patients (98%) and consisted of headache in 43 (72%), cranial nerve palsy in 33 (55%), confusion in 10 (17%), seizures in 7 (12%), and focal neurologic signs in 9 (15%). Fever and weight loss were present in 8 (13%) and 13 cases (22%), respectively. Cerebral venous thrombosis was present in 8 cases (13%). Analysis of cerebrospinal fluid showed moderate hyperproteinorachia (median 0.68 g/L; IQR 0.46–3.2) with or without pleiocytosis. Diagnosis included idiopathic pachymeningitis (n = 18; 30%); granulomatosis with polyangiitis (n = 13; 17%); Erdheim-Chester disease (n = 10; 17%); IgG4-related disease and tuberculosis (n = 3; 5% each); Rosai-Dofman disease, microscopic polyangiitis, and sarcoidosis (n = 2, 3% each); cryptococcal meningitis, Lyme disease, ear-nose-throat infection, postlumbar puncture, low spinal-fluid pressure syndrome, and lymphoma (n = 1 each). We found no difference in demographics and neurologic presentation among idiopathic pachymeningitis, Erdheim-Chester disease, and granulomatosis with polyangiitis. In contrast, frequencies were lower with idiopathic pachymeningitis than Erdheim-Chester disease for general signs (6% and 40%, respectively, *P* = .041) and complete neurologic response (0% vs 39%, *P* = .045).

The detection of extraneurologic signs and routine screening are needed to classify the pachymeningitis origin. Prospective studies are warranted to determine the best treatment in each case.

**Key Points**

- Idiopathic pachymeningitis remains the most frequent etiology of pachymeningitis.
- Routine plasma IgG4 and staining are necessary as IgG4 syndrome could mimic idiopathic pachymeningitis.
- Extraneurologic signs are important to evoke associated etiologies of pachymeningitis.

## 1 Introduction

Pachymeningitis is a rare inflammatory disease characterized by local or diffuse spinal dura thickening, which could be a feature of various conditions.^[1]^ Clinical manifestations depend on the location of inflammatory lesions and compression of adjacent nervous structures. Clinical and radiologic signs are not specific to pachymeningitis and the presence of extraneurologic features as well as immunologic, histologic, and infectious work-up could help to determine the underlying etiology. Among the main associated diseases are the infectious diseases such as tuberculosis, syphilis, cryptococcal infection, and Lyme disease; autoimmune or inflammatory diseases such as granulomatous with polyangiitis (GPA), sarcoidosis, and recently described IgG4-related disease; and malignancies, in particular lymphoma.^[1,2]^ Several case series of pachymeningitis have been reported, but large series comparing characteristics by etiologies and outcome are still lacking.^[1,3–5]^

Early diagnosis and treatment are crucial in preventing neurologic damage. Treatment mostly includes steroids or immunosuppressive therapy, but the best treatment regimen and outcome related to the various underlying situations are not determined.

In this nationwide study in France, we report the characteristics, treatment, and outcome of 60 patients with pachymeningitis and compare the profile and outcome of pachymeningitis with different etiologies.

## 2 Patients and methods

We conducted a retrospective study of patients from 17 referral centers in the “Société National Francaise de Médecine Interne” (SNFMI) network in France between March 2010 and April 2016. Physicians were asked by SNFMI to declare all cases with pachymeningitis during 12 months and data were collected retrospectively from the physicians in charge of the patients. Pachymeningitis was defined as focal or diffuse thickening of dura mater visualized on magnetic resonance imaging (MRI) of the brain and/or histologic analysis of dura mater consistent with persistent inflammation.

Clinical, laboratory, and imaging data were collected, as were data on treatments at baseline, at 6, 12 months, and at last available visit. Clinical data recorded included the presence of general symptoms; noninfectious fever; impaired lung, ear-nose-throat (ENT), kidney, nervous system, skin, joint, eye, and heart; and presence of venous/arterial thrombosis. Neurologic impairment data collected included headaches, localized neurologic signs, cranial nerve involvement, confusion, epilepsy, and vestibular and cerebellar impairment. Laboratory data recorded included proteinorachia, cerebrospinal fluid (CSF) cell numbers and type, antinuclear antibodies, antineutrophil cytoplasmic antibodies (ANCAs), ECA, syphilis serology, BK cultures from different sites if available, cryptococcal infection analysis, Borrelia analysis, HIV and human T-cell lymphotropic virus-1 serologies. Histology findings from biopsies were recorded if available, as were IgG4 immunostaining data and blood levels. Data for routine laboratory variables of disease activity, including erythrocyte sedimentation rate (ESR) and C-reactive protein (CRP) levels were collected. The presence of parenchymal impairment and ventricular dilatation were recorded on MRI.

The underlying disease was diagnosed by using international criteria (ie, American College of Rheumatology criteria for GPA), and idiopathic pachymeningitis was considered in the absence of associated disease. Steroid amount was analyzed at the initiation of each new treatment regimen and during follow-up. Neurologic treatment response was defined as complete with disappearance of all signs (clinical and/or CSF if realized) present at baseline, partial with more than 50% improvement, and nonresponse for all remaining cases. Treatment response was analyzed for extraneurologic impairments. Radiologic response was defined as complete disappearance of pachymeningitis, partial response with at least 50% decrease of thickening, and no response for all remaining cases. Steroid dependence was defined as prednisone-equivalent amount >20 mg/d for at least 2 months. Relapse was defined as reappearance of neurologic and/or extraneurologic signs after at least 3 months’ remission. The follow-up was considered from the diagnosis time to the last available news.

The study was performed in accordance with the ethical standards of the Helsinki Declaration and was approved by an institutional review board (Comité de Protection des Personnes, Aulnay sous Bois, Ile de France X).

### 2.1 Statistical analysis

Data are described with mean ± standard deviation and median (interquartile range [IQR]) for continuous variables and frequencies (%) for categorical variables. Results are expressed as observed data (missing data not replaced) to account for nonavailable data and percentages are calculated take into account the overall number of available cases. Chi-squared or Fisher exact tests were used to compare categorical variables and Mann–Whitney *U* or Student *t* test to compare continuous variables. All tests were 2 sided and *P* < .05 was considered statistically significant. Statistical analyses involved use of GraphPad v3.1.0.

## 3 Results

### 3.1 Baseline characteristics of patients

We included 60 patients (median age 55.5 years; IQR 30–80, female/male ratio 0.43) (Table 1). Median ESR and CRP levels were 32 mm (IQR 10–131) and 10 mg/L (IQR 1–200), respectively (Table 1). CSF analysis showed moderate hyperproteinorachia (0.68 g/L, IQR 0.46–3.2), and cytologic findings were heterogeneous, with more than half of the patients showing normal cell count (<10 cells/mm^3^: n = 18; 53%). The remaining patients showed lymphocytic meningitidis (median 7 cells, IQR 10–360). MRI of the brain showed pachymeningitis in all patients, with a diffuse pattern in 19 (34%), ventricular dilatation in 4 (10%), and parenchymal extension in 11 cases (20%). Cerebromeningeal biopsy was performed in 18 patients (30%), showing mainly nonspecific inflammatory reaction and lymphoplasmacytic infiltrates in 6 cases. Three of 20 patients with cerebral and/or salivary-gland biopsy involving IgG4 staining had positive staining. The final diagnosis was idiopathic pachymeningitis (n = 18; 30%), GPA (n = 13; 17%), Erdheim-Chester disease (n = 10; 17%), IgG4-related disease and tuberculosis (n = 3 each; 5%), Rosai-Dofman disease, microscopic polyangiitis and sarcoïdosis (n = 2 each; 3%), and cryptococcal meningitis, Lyme disease, ENT infection, postlumbar puncture, low spinal fluid pressure syndrome, and lymphoma (n = 1 each). The median time from first symptoms to diagnosis was 7 months (IQR 0–300).

**Table 1:**
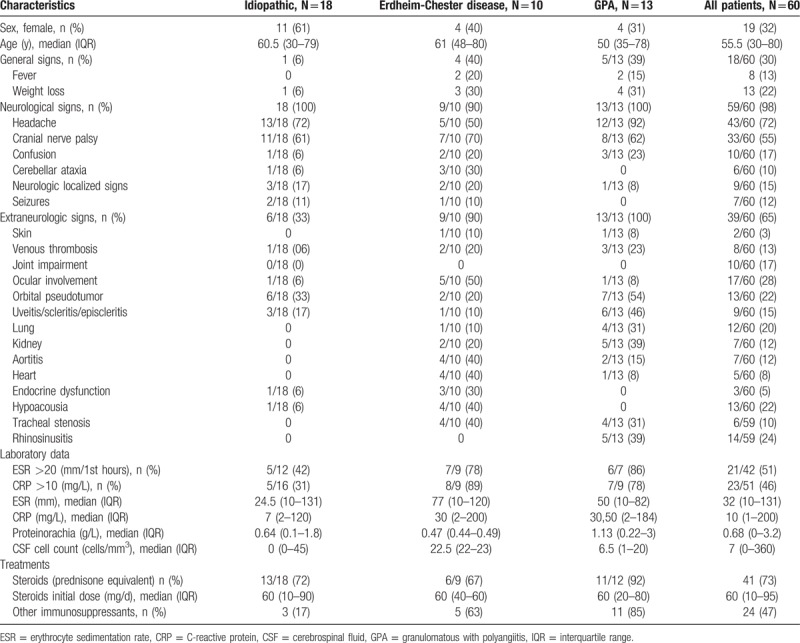
Patients’ characteristics and treatments according to etiology (data are expressed as median and percentages considering the missing data).

### 3.2 Comparison of idiopathic pachymeningitis, Erdheim-Chester disease, and GPA

For the 18 patients with idiopathic pachymeningitis, the median age was 60.5 years (IQR 30–79; female/male ratio 0.61) (Table 1). All patients had neurologic signs, mainly headache (n = 13; 72%) and cranial nerve impairment (n = 11; 61%). Six patients (33%) showed extraneurologic signs, including orbital pseudotumor (n = 6) and uveitis (n = 3); 1 patient with cavernous venous thrombosis. Elevated ESR (>20 mm) and CRP level (>10 mg/L) were noted in 5/12 patients (42%) and 5/16 (31%), with median proteinorachia 0.64 g/L (IQR 0.1–1.8) and cell count 0 cells/mm^3^ (IQR 0–45). MRI revealed diffuse pachymeningitis in 5 patients (42%), without any ventricular dilatation. Steroids were used in 13/18 patients (72%) with an initial median dose of 60 mg/d (IQR 10–90).

Ten patients had Erdheim-Chester disease (median age 61 years; IQR 48–80, female/male ratio 0.4) (Table 1). Neurologic signs occurred in 9 patients (90%), mainly cranial nerve impairment (n = 7; 70%) and headache (n = 5; 50%); only 2 patients had localized neurologic signs. Extraneurologic signs were present in 9/10 patients (90%) and mainly consisted in perivascular impairment (n = 4; 40%), hypoacousia (n = 4; 40%), and kidney involvement (n = 2; 20%). Elevated ESR (>20 mm) and CRP level (>10 mg/L) were noted in 7/9 (78%) and 8/9 (89%) patients, respectively. Median proteinorachia was 0.47 g/L (IQR 0.44–0.49) and cell count 22.5 cells/mm^3^ (IQR 22–23). MRI revealed diffuse pachymeningitis in 3 patients (30%), without any ventricular dilatation. Steroids were used in 6/9 patients (67%) with an initial median dose of 60 (IQR 40–60) mg/d.

Thirteen patients had GPA (median age 50 years, IQR 35–78, female/male ratio 0.3) (Table 1). Neurologic signs were found in all patients, mainly cranial nerve impairment (n = 8; 62%) and headache (n = 12; 92%). Extraneurologic signs were present in all patients and consisted mainly of orbital pseudotumor (n = 7; 54%), uveitis (n = 6; 46%), tracheal stenosis (n = 4; 31%), and hypoacousia (n = 4; 31%). Lung involvement was noted in 4 patients (31%) and kidney involvement in 5 (39%). Elevated ESR and CRP level was noted in 6/7 (86%) and 7/9 (78%) patients, respectively. Median proteinorachia was 1.13 g/L (IQR 0.22–3) and cell count 6.5 cells/mm^3^ (IQR 1–20). MRI showed diffuse pachymeningitis in 3 patients (23%), 1 with ventricular dilatation. ANCAs were present in all patients, with anti-PR3 specificity in 7/11 (64%). Steroids were used in 11/12 patients (92%) with an initial median dose of 60 mg/d (IQR 20–80). Associated immunosupressive drugs were cyclophosphamide (n = 7), rituximab (n = 2), methotrexate (n = 1), and mycophenolate mofetil (n = 1).

Because other etiologies were not sufficiently represented, we compared patients with idiopathic pachymeningitis, Erdheim-Chester disease, and GPA and found no difference in age, sex, frequency and type of neurologic signs, and extension of pachymeningitis. Frequency of general signs was lower in idiopathic pachymeningitis than Erdheim-Chester disease (6% and 40%, *P* = .041) and that of extraneurologic symptoms was lower in idiopathic pachymeningitis than Erdheim-Chester (33% vs 90%, *P* = .006), mainly ocular (6% vs 50%, *P* = .0126), kidney (0% vs 20%, *P* = .041), and aortitis (0% vs 40%, *P* = .02) (Table 1). Elevated CRP level was more frequent with Erdheim-Chester disease than idiopathic pachymeningitis (78% vs 42%, *P* = .012). Median proteinorachia was similar with the 2 diseases, but increased number of CSF cells was greater with Erdheim-Chester disease (22.5 [IQR 22–23] vs 0 [0–45] cells/mm^3^, *P* = .05).

The frequency of general signs was greater with GPA than idiopathic pachymeningitis (40% vs 6%, *P* = .04), and extraneurologic symptoms were more frequent (100% vs 33%, *P* = .0001), in particular tracheal stenosis (31% vs 0%, *P* = .008) and rhinosinusitis (39% vs 0, *P* < .0001), with more frequently elevated ESR (86% vs 42%, *P* = .041). Median proteinorachia was similar between the diseases; CSF pleiocytosis tended to be higher with GPA (6.5 [IQR 1–20] vs 0 [0–45] cells/mm^3^; *P* = .07).

### 3.3 Outcome

Immunosuppressive treatment was initiated in 56 patients and consisted of steroids for 41 (73%), with median dose 60 (IQR 10–95) mg/d; cyclophosphamide (n = 11), azathioprine (n = 5), methotrexate (n = 4), and ciclosporin, interferon, and rituximab (n = 3 each) (Table 1). Patients with tuberculosis (n = 3) received antituberculosis drugs, associated with steroids for 2 cases. Ceftriaxone was used for 3 weeks with Lyme disease. Surgical intervention was needed for 9 patients (17%), mainly for ventricular dilatation.

Among 56 treated patients, 41 were evaluated at 6 months and 48 at the last visit during a median follow-up of 4 years (IQR 1–9) (Table 2). Neurologic response was complete and partial at 6 and 12 months for 14 (34%) and 10 (24%), and 9 (27%) and 8 (24%) patients, respectively (Table 2). Daily steroids dose significantly decreased after 12 months of treatment (median 60 mg [IQR 10–95] at baseline vs 15 mg [5–90] at 12 months, *P* < .001). At 6 months, MRI complete response was noted in 7 patients (19%), with partial regression in 22 (61%).

**Table 2:**
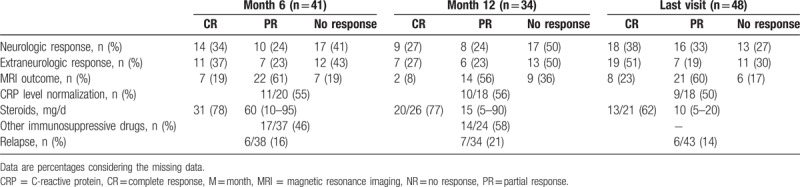
Outcome and with treatment by response (complete, partial, or no response) at 6 and 12 months and last visit.

With idiopathic pachymeningitis, at 6 and 12 months, neurologic partial and complete response were observed in 5 (39%) and 4 (31%), and 4 (40%) and 3 (30%) patients, respectively. MRI partial response was observed in 5/8 patients (63%). With a median follow-up of 6 years (IQR 1–13), at the last visit, 9/14 patients showed neurologic partial or complete response, with MRI complete or partial response in 7/9 (78%). Steroids were still used in 9/12 patients at 6 months (75%).

With Erdheim-Chester disease, at 6 and 12 months, neurologic partial or complete response was found for 1 (20%) and 0 and 1 (20%) and 0 patients, respectively. MRI partial response was noted in 3/5 patients (60%). Three of 5 patients at the last visit showed neurologic partial or complete response, with MRI partial response in 4/5 (80%).

With GPA, at 6 and 12 months, neurologic partial/complete response was found for 3 (33%) and 2 (22%) patients, respectively. MRI partial response was noted in 3/4 (75%) patients. Ten of 13 patients at the last visit showed neurologic partial or complete response, with MRI partial response in 8/9 (89%). Steroids were still used in 8/9 patients at 12 months (89%).

Neurologic partial and complete response were similar at 6 months for patients with GPA and idiopathic pachymeningitis (33% and 22% vs 31% each with idiopathic pachymeningitis). Neurologic complete response was more frequent with idiopathic pachymeningitis than Erdheim-Chester disease (39% vs 0%, *P* = .045). The frequency of patients under steroid treatment at 6 months was similar with idiopathic pachymeningitis (82%), GPA (100%), and Erdheim-Chester disease (83%). At 6 months, the use of immunosuppressive therapy was more frequent with Erdheim-Chester disease (67%) and GPA (100%) than idiopathic pachymeningitis (10%; *P* = .357 and *P* = .0001, respectively).

**Figure 1:**
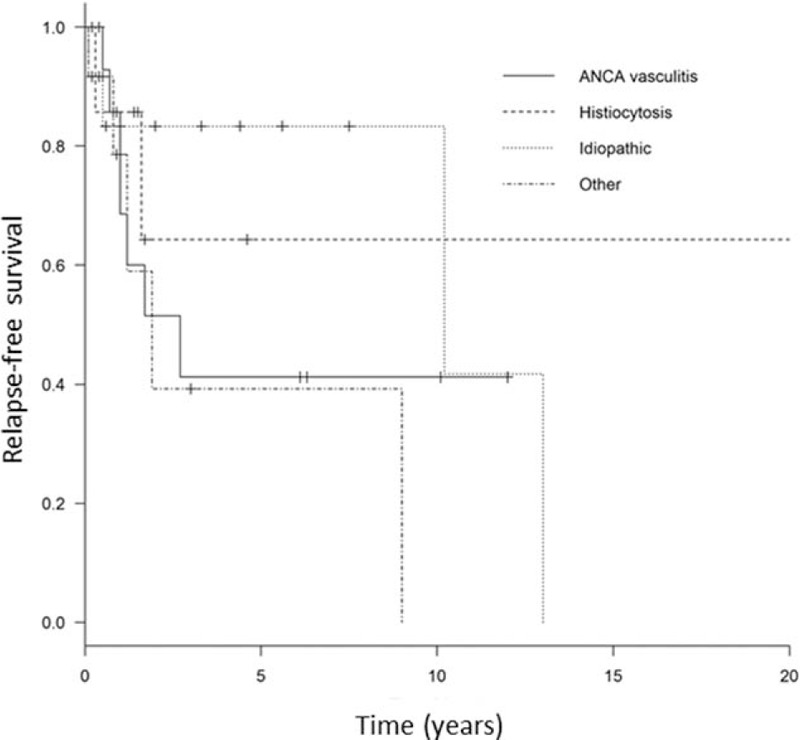
Relapse-free survival in antineutrophil cytoplasmic antibody (ANCA)-associated vasculitis, Erdhem-Chester disease, and idiopathic forms.

Relapse-free survival significantly differed among these 3 diseases, with median time to relapse to 2.7 years in patients with GPA and 10.2 years in idiopathic pachymeningitis and was not reached in Erdheim-Chester disease (log rank *P* < .05) (Fig. 1).

## 4 Discussion

We report here one of the largest series of pachymeningitis including descriptions and for the first time the comparison of features, response rates, and outcome of several etiologies of pachymeningitis. Idiopathic pachymeningitis represented the most frequent situation, and several extraneurologic signs, particularly ENT and ocular involvements, should be screened and can help pinpoint the etiology. Acute-phase reactants, immunologic data, serum IgG4 dosage, and infectious work-up are needed to prevent misdiagnosing the underlying disease, in particular with only isolated neurologic signs. The outcome is still challenging, because 30% of patients have persistent signs despite treatment. In this study, we show the particular different relapse-free survival according the underlying disease.

In our case series, one of more common entities remained idiopathic pachymeningitis (30%), which is similar to previous data.^[3,4]^ A survey in Japan revealed idiopathic pachymeningitis in 44% of cases, followed by ANCA-associated vasculitis and in particular GPA.^[4]^ Idiopathic pachymeningitis is a challenging diagnosis, and its characteristics are similar to an emerging inflammatory condition, the IgG4-related syndrome. Only a few data are available to determine the part of IgG4-related syndrome among “idiopathic pachymeningitis.”^[2,6,7]^ Wallace et al performed IgG4 immunostaining for 14 cases of idiopathic pachymeningitis and reclassified 4 cases (29%) as IgG4 syndrome,^[7]^ which emphazises the importance of this analysis in all cases of idiopathic pachymeningitis, even isolated cerebral forms.

Few data are available concerning pachymeningitis in GPA and other ANCA-associated vasculitis.^[3,8–12]^ One of the recent important series analyzed central nervous system involvement in GPA and found a 46% prevalence of pachymeningitis, defining a granulomatous pattern.^[11]^ These patients commonly have headaches and more frequently myeloperoxidase-ANCA, which differs from our series, 64% with PR3 specificity. In our GPA patients, pachymeningitis was frequently associated with orbital pseudotumor, tracheal stenosis, and rhinosinusitis and less frequently, a kidney and lung pattern. In Erdheim-Chester disease, 10% to 25% of patients had neurologic involvement, a factor associated with disease severity.^[13–15]^ The neurologic involvement is rarely isolated, and the presence of extraneurologic features could help in the diagnosis.

Few studies have compared the clinical features and outcome of patients with idiopathic pachymeningitis and other pachymeningitis diseases. Among 28 patients, 20 had idiopathic pachymeningitis and no specific neurologic, CSF data or MRI features in comparison to secondary pachymeningitis.^[16]^ Another study comparing idiopathic pachymeninigitis to diseases related to ANCA-associated vasculitis and IgG4 syndrome showed a predominant male ratio in idiopathic forms and older age in GPA patients.^[4]^ In our study, the presence of general signs, extraneurologic signs, high CSF cell number and acute-phase reactants allowed for discriminating idiopathic disease from other etiologies.

Our study contains several limitations. All consecutive patients could not be included in this study, and thus the frequency of various pathologies is not representative of their real prevalence. As well, many patients were selected from internal medicine departments, which could underrepresent some etiologies such as infectious disease. The few cases of pathologies such as sarcoidosis, paraneoplastic, and infectious diseases did not allow us to evaluate the features and outcome of pachymeningitis associated with these disorders. In idiopathic pachymeningitis, IgG4 serum levels and IgG4+ plasmocyte analysis were not always available, because some cases were diagnosed before this analysis could be performed and thus could not be excluded as misdiagnosis. The absence of IgG4 screening in all cases could underestimate the real prevalence of this entity in the subset of pachymeningitis.

## 5 Conclusion

The characteristics and the etiologies of pachymeningitis need to be carefully analyzed. Routine screening and detection of extraneurologic signs are needed to classify the pachymeningitis origin. Prospective studies are needed to determine the best treatment in each case.

## Author contributions

Study concept and design: AM, LM, OF.

Acquisition of data: AM, LM, OF, LM, LB, CM, NA, EG, FL, RC, RRW, LD, CM, BT, XA, NS, MH, AK, PG, FL, ZA, JH, CL, JFB, SH, JBF, JCP, LG, RD, JH.

Analysis and interpretation: AM, LM, OF, LM, LB, CM, NA, EG, FL, RC, RRW, LD, CM, BT, XA, NS, MH, AK, PG, FL, ZA, JH, CL, JFB, SH, JBF, JCP, LG, RD, JH.

Critical revision of the manuscript for important intellectual content: AM, LM, OF, LM, LB, CM, NA, EG, FL, RC, RRW, LD, CM, BT, XA, NS, MH, AK, PG, FL, ZA, JH, CL, JFB, SH, JBF, JCP, LG, RD, JH.

Study supervision: OF -.

**Conceptualization:** Arsene Mekinian, Lucas Maisonobe, Latifatou Boukari, Clea Menotte, BENJAMIN Terrier, Xavier Ayrignac, Nicolas Schleinitz, Damien Sene, Mohamed Hamidou, Amadou Konate, Philippe Guilpain, Noemie Abisror, Etienne Ghrenassia, Florence Lachenal, Ramiro Cevallos, Richard Roos, Du Boutin, Francois Lhote, Claire Larroche, Jean Bergmann, Sebastien Humbert, Jb Fraison, Jean Piette, Loic Guillevin, Robin Dhote, Zahir Amoura, Julien Harroche, Olivier Fain.

**Data curation:** Arsene Mekinian, Lucas Maisonobe, Latifatou Boukari, Clea Menotte, Benjamin Terrier, Xavier Ayrignac, Nicolas Schleinitz, Damien Sene, Mohamed Hamidou, Amadou Konate, Philippe Guilpain, Noemie Abisror, Etienne Ghrenassia, Florence Lachenal, Ramiro Cevallos, Richard Roos, Du Boutin, Francois Lhote, Claire Larroche, Jean Bergmann, Sebastien Humbert, Jb Fraison, Jean Piette, Loic Guillevin, Robin Dhote, Zahir Amoura, Julien Harroche, Olivier Fain.

**Formal analysis:** Arsene Mekinian, Lucas Maisonobe.

**Funding acquisition:** Arsene Mekinian, Lucas Maisonobe.

**Investigation:** Arsene Mekinian, Lucas Maisonobe.

**Methodology:** Arsene Mekinian, Lucas Maisonobe.

**Project administration:** Arsene Mekinian, Lucas Maisonobe.

**Resources:** Arsene Mekinian, Lucas Maisonobe.

**Software:** Arsene Mekinian, Lucas Maisonobe.

**Supervision:** Arsene Mekinian.

**Validation:** Arsene Mekinian, Lucas Maisonobe.

**Visualization:** Arsene Mekinian, Lucas Maisonobe.

**Writing – original draft:** Arsene Mekinian, Lucas Maisonobe.

**Writing – review & editing:** Arsene Mekinian, Lucas Maisonobe.
