# Characteristics, outcome and treatments with cranial pachymeningitis: A multicenter French retrospective study of 60 patients: Erratum

**DOI:** 10.1097/MD.0000000000012063

**Published:** 2018-08-17

In the article, “Characteristics, outcome and treatments with cranial pachymeningitis: A multicenter French retrospective study of 60 patients”,^[1]^ which appeared in Volume 97, Issue 30 of *Medicine*, Dr. Nicolas Schleinitz's name appeared incorrectly as Nicolas Scheinlitz.
